# A Reflective Thematic Analysis Into the Perceptions of Pregnant Radiographers Regarding the Usefulness of the PregiDose Mobile App to Enhance Fetal Dosimetry and Well-Being: Qualitative Exploration

**DOI:** 10.2196/58608

**Published:** 2024-11-15

**Authors:** Hafsa Essop, Ramadimetja Mable Kekana, Jacques Brosens, Hanlie Smuts

**Affiliations:** 1 Department of Radiography University of Pretoria Pretoria South Africa; 2 Department of Informatics University of Pretoria Pretoria South Africa

**Keywords:** mobile app, design science research, usefulness, pregnant radiographers, fetal dosimetry, occupational health and safety, mobile phone, maternal and child health, PregiDose

## Abstract

**Background:**

Pregnancy apps are widely used by pregnant women, who benefit from self-tracking features to support their health goals. Pregnant radiographers are considered a high-risk group of health workers practicing in ionizing radiation environments. Radiation exposure above threshold limits can cause harmful genetic effects on a fetus. Accordingly, pregnant radiographers are required to wear special fetal dosimeters, which provide real-time readings of radiation dose exposure to the fetus. Pregnant radiographers have the responsibility to self-track their fetal doses to ensure that the threshold limit of 1 mGy is not exceeded. The traditional method used to track doses includes a written log of doses in a notebook. Thus, PregiDose, a unique offering in the context of pregnancy apps, was developed to enhance fetal dose tracking and monitoring using technological methods.

**Objective:**

This study aims to describe the users’ perceptions of the app’s usefulness using PregiDose in a natural setting.

**Methods:**

The overarching framework adopted for the study was a design science research (DSR) methodology encompassing five steps, namely (1) problem awareness, (2) suggestion, (3) development, (4) evaluation, and (5) conclusion. This paper presents the evaluation step of DSR. DSR step 4 included a qualitative approach to explore users’ perceptions regarding the app. Data were collected using a semistructured interview guide. Open-ended questions were guided by the app’s core features, namely dose tracking, education, and wellness. In total, 17 pregnant radiographers in South Africa enrolled to use the app, 9 (53%) engaged with the app, and 4 (24%) agreed to participate in the feedback interviews. The data were collected from October 2023 to March 2024 and analyzed using a reflective thematic data analysis method.

**Results:**

Three overarching themes emerged from the data, namely (1) usefulness of PregiDose, (2) barriers to PregiDose adoption and use, and (3) recommendations for the advancement of PregiDose. Users labeled the app’s usefulness as positive and perceived it as a modern approach to traditional dose-tracking methods. They perceived the graph output of the dose-tracking feature to be useful for viewing their accumulative doses. They did not fully engage with the journaling feature, indicating that it was a personal preference and not a practice they would usually engage in. Physiological barriers, such as fatigue and “pregnancy brain,” were contributors to decreased engagement. Finally, because of the demanding workload and fast-paced nature of the radiography department, users recommended the automation of fetal dosimetry through the Internet of Things.

**Conclusions:**

PregiDose is an occupational health and safety mobile app developed for pregnant radiographers through a DSR approach. The app offers a modern method of dose tracking consistent with technological advancements in the context of self-tracking. However, future implementation would require using Internet of Things to make fetal dose tracking more effective.

## Introduction

### Background

The adoption of mobile apps, facilitated through smartphones, is rapidly increasing because of the ease of access to smartphones and people’s dependence on technology [1,2]. Smartphone mobile apps can support human needs by monitoring daily activities [1]. Such monitoring provides insights into the daily activities of an individual to prevent and manage acute or chronic diseases [3]. There is a growing emphasis on individual health surveillance for preventive health care. Interacting regularly with their data enables users to identify trends and early warnings for health-related issues. This facilitates proactive interventions toward preventing severe health conditions. Therefore, mobile apps are at the center of successful technology adoption, coupled with the widespread availability of smartphones in both low- and middle-income countries and high-income countries [2].

Pregnant women, specifically, are moving toward using technology as a means of support throughout their pregnancies [1]. Lupton and Pedersen [4] describe three main categories of pregnancy apps, namely (1) entertainment, (2) self-monitoring, and (3) pregnancy education. These categories enable pregnant women to closely monitor their health and that of their fetuses, and gain knowledge and understanding of the different stages of the pregnancy. Furthermore, these factors contribute to reassuring the mother of the unborn child about their child’s health and well-being [4]. The World Health Organization places great importance on individuals’ good health and well-being, widely regarded as Sustainable Development Goal 3. Pregnant women undergo somatic, physiological, and emotional changes during pregnancy, which often increase their stress levels, thereby impacting the fetus [5]. The World Health Organization describes maternal health as a woman’s good health and well-being during pregnancy, whereby each stage is a positive experience, enabling both mother and child to thrive [6].

Pregnant radiation workers, like radiographers, work in ionizing radiation environments that can potentially be harmful to the developing fetus. Various levels of radiation exposure can cause damage to cells and tissues and are classified as either deterministic or stochastic [7]. Deterministic effects are the result of excessively high radiation exposures [7]. The effects of high radiation doses on a fetus were documented through the Chernobyl and Fukushima events. Exposures exceeding 100 mSv resulted in miscarriage, fetal malformations, neurobehavioral abnormalities, fetal growth retardation, and cancer [8]. Stochastic effects include nonspecific and nonpredictable effects that can cause DNA alteration, leading to genetic mutations or cancer [7]. For a pregnant woman, both types of effects, particularly stochastic effects, can evoke fear and anxiety [9]. The fear of radiation itself has the potential to threaten the health and well-being of the fetus through maternal stressors [10].

Occupational health and safety regulations for pregnant radiographers include wearing a fetal dosimeter and recording fetal exposure [11]. The International Commission for Radiological Protection recommends a threshold limit of 1 mGy, which is considered safe and has negligible deterministic effects on the fetus [11]. Therefore, the radiation worker is responsible for ensuring that the dose does not exceed the threshold limit by monitoring fetal exposure daily. However, a study by Essop et al [12] reveals that uptake of these recordings and monitoring measures remains low. Thus, it is evident that pregnant radiation workers are a high-risk group of women with requirements beyond that of pregnant women in other occupations. Mobile apps for pregnant women are widely available; however, in light of the unique considerations for pregnant radiographers, there is a need to harness technology to provide a tailored intervention regarding inconsistent fetal dose monitoring. Accordingly, the researchers in this study developed a mobile app called PregiDose through a design science research (DSR) strategy.

**Figure 1:**
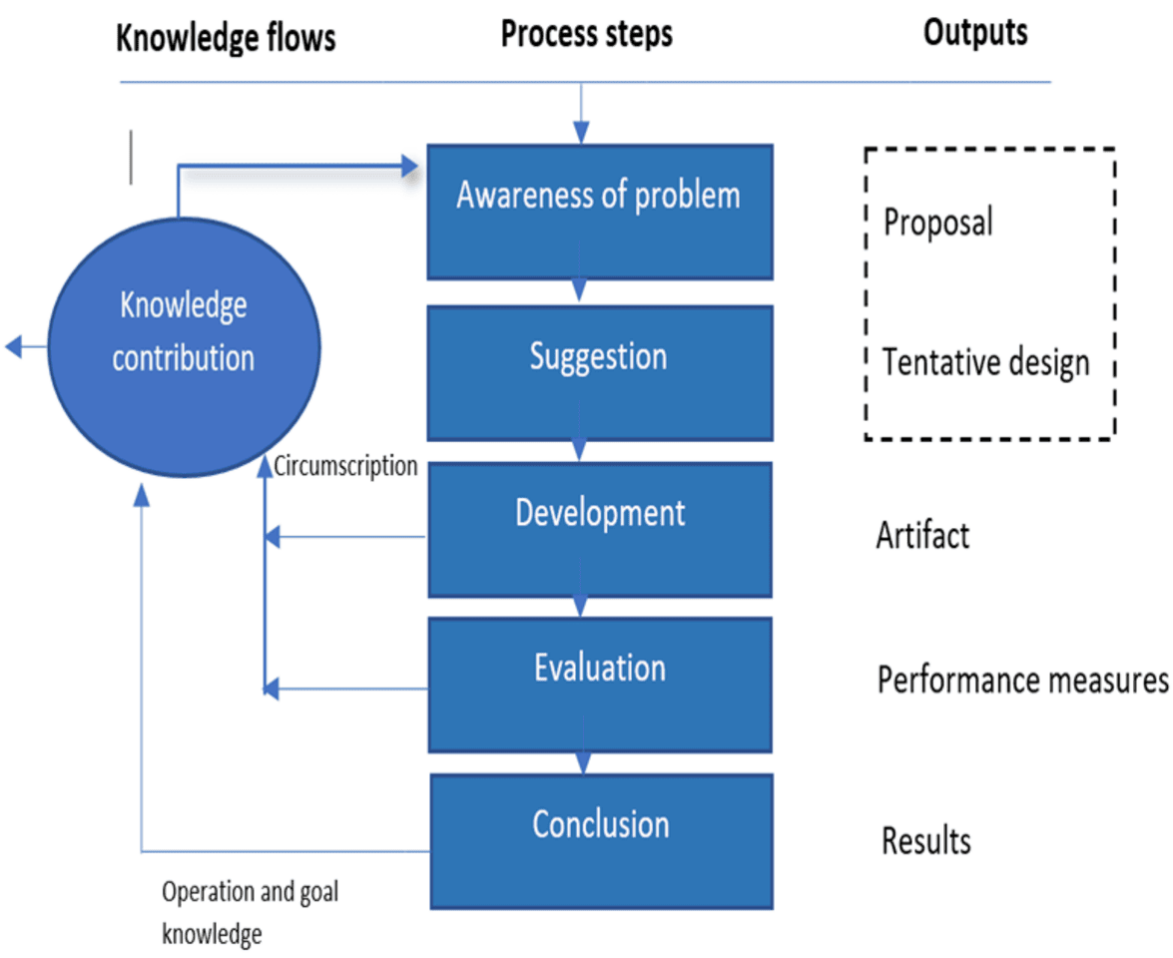
Design science research process by Vaishnavi and Kuechler [19] used to inform the overarching methodology for this study.

DSR is a widely accepted research paradigm for developing mobile apps in information systems, as it is used to create and evaluate artifacts that do not yet exist [13]. These artifacts are unique inasmuch as they are specifically aimed at rigorously solving organizational problems emanating from the environment. Hence, problems emanating from these contexts should be conceptualized and constructed properly with appropriate methods for finding a solution [14]. The final artifact should be evaluated to ensure it complies with the criteria for a DSR artifact [14]. March and Smith [14] described four historical outputs of DSR: (1) constructs, (2) models, (3) methods, and (4) instantiations. Instantiations refer to the realization of an artifact in its environment. Examples of artifacts that have successfully adopted the DSR paradigm to guide mobile app development include apps for people living with HIV [15], blockchain in health care [16], and several other mobile health (mHealth)-related apps [17]. One of the first implementations of the DSR framework in radiography was successfully used to develop an artifact for computer-aided detection for chest pattern recognition training [18]. DSR typically uses a systematic process encompassing a set of well-accepted guidelines [13]. Vaishnavi and Kuechler [19] developed a widely used process to execute DSR research and explained the five steps in the process, namely (1) awareness of the problem, (2) suggestion, (3) development, (4) evaluation, and (5) conclusion (Figure 1 [19]).

### This Study

This study aimed to provide an overview of the DSR process used to guide the development of a mobile app and present the final evaluation phase of the DSR process.

## Methods

### Overview

**Figure 2:**
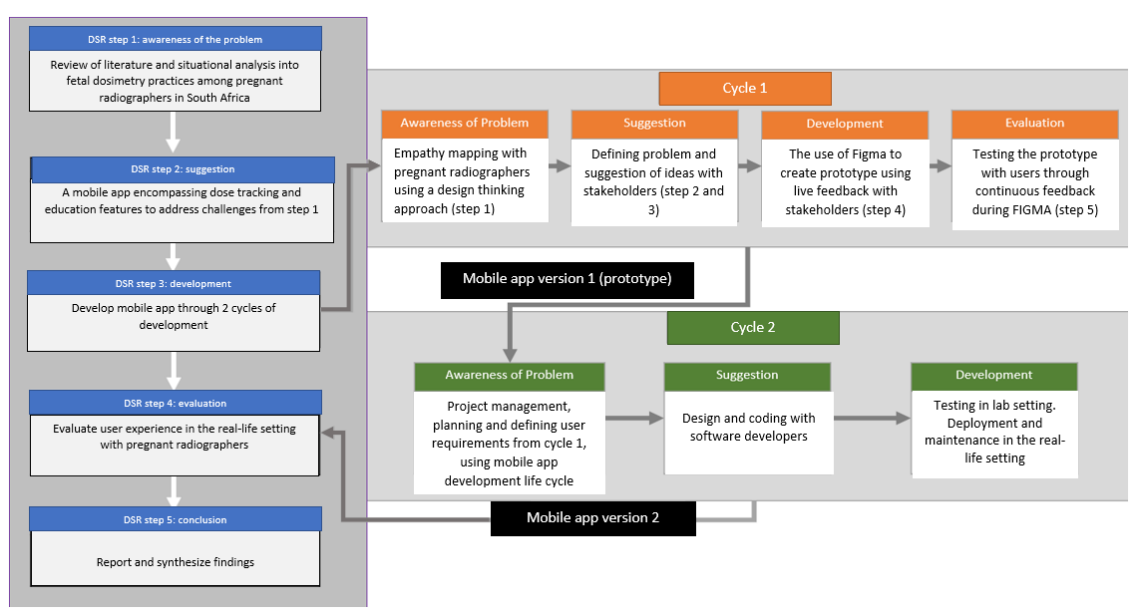
Summary of the 5 keys steps of the design science research process used to develop and evaluate the app.

The study followed the DSR process proposed by Vaishnavi and Kuechler [19] to guide each research step. The researchers modified the process to reflect the variables related to the study in terms of the specificities of the methods used in each step (Figure 2).

Step 1 included a survey of 89 pregnant and previously pregnant radiographers from January 2022 to April 2022. The goal of this step was to ascertain the methods used to monitor fetal doses and identify challenges in the context of occupational health and safety among pregnant radiographers [12]. In step 2, results from the survey led to the suggestion phase of the study, whereby, a suggestion was made on the appropriate technological solution for the identified problem. Two cycles of development took place in step 3 (Figure 2). In cycle 1, a user-centered approach informed the design of the prototype [20]. In January 2023, a participatory design workshop was conducted with a multidisciplinary group of 12 participants in a focus group setting. This included pregnant radiographers, previously pregnant radiographers, quality assurance officers, medical physicists, managers, and radiation regulators. The stakeholders were taken through 5 steps of design thinking, namely empathy, define, ideate, prototype, and testing. The cycle adopted a qualitative approach. A specification document was compiled from the data which were provided to the software developers in cycle 2 [20]. In cycle 2, the software developers used a mobile app development life cycle model that encompassed eight steps as follows: (1) project management and planning, (2) requirements, (3) design, (4) coding, (5) testing, (6) deployment, (7) maintenance, and (8) projection evaluation [21]. The team consisted of a project manager, a user experience designer, a user interface designer, 2 Flutter developers, and a test analyst. The entire development process was executed over 5 months (August 2023 to January 2024; Figure 2).

In step 4, the end users, namely pregnant radiographers, engaged with the app in a real-life setting for 6 months. Thereafter, the researcher conducted qualitative interviews with the users to explore and describe their perceptions regarding the mobile app’s usefulness. Finally, in step 5, the overall findings of the study were synthesized and evaluated against the DSR criteria for developed artifacts.

### App Description

**Figure 3:**
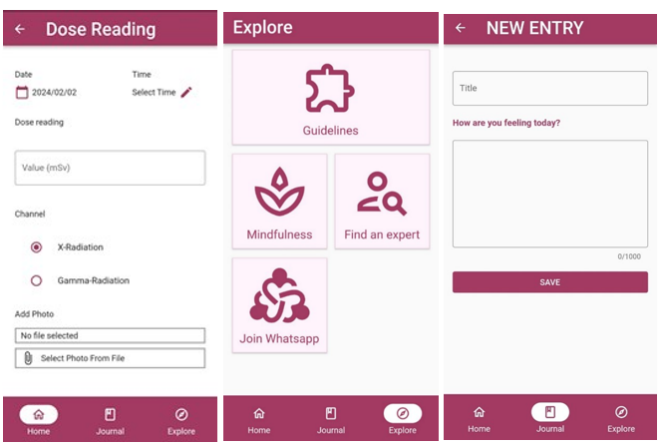
Excerpts of the PregiDose app features, including, dose-tracking input, educational links for pregnant radiographers, guidelines, mindfulness practices, and social support. Finally, the daily journaling entry feature is also shown.

PregiDose is a mobile app for pregnant radiographers intended to support fetal dose monitoring and the mental well-being of radiographers (Multimedia Appendix 1). The app’s core feature includes a dose-tracking feature, which enables pregnant radiographers to input their daily fetal doses from their dosimeter and monitor their accumulative doses to ensure fetal doses do not exceed the threshold limit of 1 mGy. Education support features include guidelines and policies specific to pregnant radiographers. Finally, the wellness features of the app include links to mindfulness practices, social support through social media groups, and journaling, whereby users can input and express their thoughts and feelings during their pregnancy. Figure 3 illustrates screenshots of these features.

### Recruitment and Sampling

The study population included pregnant radiographers working in diagnostic, radiation therapy, and nuclear medicine departments in South Africa. Pregnant women working in these departments who did not have access to a dosimeter were still included in the study, as they would be able to engage with other features of the app not related to the dose-tracking function. The exclusion criteria were pregnant radiographers using iOS mobile devices, which were not yet supported on the mobile app platform, as well as pregnant radiographers who were not working within ionizing radiation departments, such as ultrasound and magnetic resonance imaging.

The participants were recruited through 2 sampling methods, namely purposive sampling followed by snowballing sampling. In purposive sampling, the database from the situational analysis phase of the study was accessed. In this phase, the women participants were given the opportunity to provide details to engage with the app should they become pregnant during the development completion. This sample only yielded 2 pregnant participants and therefore required the second sampling method to be used. In the snowballing sampling, the researcher sent out an invitation link to her professional circle and heads of departments in selected clinical training hospitals in South Africa. Finally, the invitation was also shared on radiography-specific social media platforms, such as Locum Radiographers South Africa and the researcher’s personal LinkedIn (LinkedIn Corporation) page with a large following of South African radiographers. The invitation link provided an overview of the study and served as informed consent upon completion. This invitation also required the contact details of the participants and the types of phones they used to screen eligible participants. The app was only available to Android (Google LLC) users at this stage of testing; therefore, iOS (Apple Inc) users were excluded from the study. Informed consent was obtained from the participants during the recruitment stage, whereby they were given information regarding the study through an information link. If they were interested in the study, they were asked to complete the web-based consent form to engage with the app and partake in feedback interviews thereafter. Only after obtaining consent, were the app made available to the participants.

### Data Collection

Suitable participants were registered on the app, and an information package, including guidelines on the use of PregiDose, an ethics certificate, and the Android package kit file, was then sent to the participants via email. Subsequently, the participants could engage with the app in a real-life setting from October 2023 to March 2024. Thereafter, they were invited to a telephonic interview to share their experiences. In total, 17 pregnant radiographers responded to the invitation and shared their details for registration. However, 9 (53%) pregnant radiographers actually engaged with the app, and only 4 (24%) responded to the interview invitation. Table 1 describes the limitations of the sample size.

**Table 1:** Database of pregnant women in South Africa who completed the invitation, downloaded the app, and participated in an interview from October 2023 to March 2024.

Participant code	Due date	Department	Completed invitation to participate in the study	Downloaded the app and registered	Access to dosimeter	Response to the telephonic interview
1	November 11, 2023	Diagnostic	Yes	No (gave birth before downloading)	—^a^	—
2	March 29, 2024	Diagnostic	Yes	Yes	No	No
3	October 5, 2023	Diagnostic	Yes	No (gave birth before downloading)	—	—
4	February 13, 2024	Diagnostic	Yes	No (no response to follow-up)	—	—
5	November 6, 2023	Diagnostic	Yes	No (no response to follow-up)	—	—
6	March 3, 2024	Diagnostic	Yes	Yes	No	No
7	April 12, 2024	Diagnostic	Yes	No (no response to follow-up)	—	—
8	June 5, 2024	Diagnostic	Yes	No (iOS phone user)	—	—
9	January 26, 2024	Diagnostic	Yes	Yes	Yes	Yes
10	February 15, 2024	Diagnostic	Yes	Yes	Yes	No
11	June 13, 2024	Diagnostic	Yes	Yes	No	Yes
12	March 6, 2024	Diagnostic	Yes	No (iOS phone user)	Yes	—
13	August 22, 2024	Diagnostic	Yes	Yes	No	No
14	April 28, 2024	Radiation therapy	Yes	Yes	Yes	Yes
15	April 23, 2024	Diagnostic	Yes	Yes	Yes	No
16	October 25, 2024	Diagnostic	Yes	Yes	Yes	Yes
17	—	Radiation therapy	Yes	No (iOS phone user)	Yes	—

^a^Information is unknown as they did not download the app, which requires information regarding access to the dosimeter.

The researcher who conducted the interviews had in-depth knowledge regarding all the features of the app. The researcher is also a woman who was pregnant while working as an academic radiographer. The telephonic interviews were guided by a semistructured interview guide, which allowed for probing questions. The researcher’s role as an interviewer was important here, as she could relate to pregnant radiographers as well as the different features that the participants were reflecting upon. The researcher did not have any relationship with the participants before the research study. The overarching research question posed to the participants was, “What are your perceptions and experiences regarding the usefulness of *PregiDose*?”

Prompting questions ensued, which aligned with the mobile app’s features, such as dose tracking and education links and wellness functions, such as mindfulness practices and journaling. Field notes, which were particularly important comments by the participants, were taken by the researcher during the interview, to refer back to during the data analysis process. The duration of the interviews was approximately 15 to 30 minutes. After each response, the researcher confirmed her understanding of the participant’s comments by summarizing them, allowing the participants to agree or disagree with the conclusion.

The interviews were recorded with a voice recorder and transcribed using an artificial intelligence–powered software, Transkriptor. The researcher evaluated the transcription to ensure accuracy and congruency with the audio recordings. The analysis was verified by the coauthors of the paper to ensure the validity and reliability of the results.

### Ethical Considerations

The study gained ethics approval from the Faculty of Health Care Sciences, University of Pretoria, Research Ethics Committee (613/2021). The participants’ autonomy was maintained by informing them that they could withdraw from the study at any time. This included both engagement with the app and being interviewed on their feedback. In this study, it was observed that most participants preferred using the app; however, only a limited number responded to the follow-up interview invitation. Nonmaleficence was maintained by ensuring that the pregnant radiographers continued to use their original, traditional methods of dose tracking in conjunction with the app, as the app was still in its testing phases of usability and usefulness. In addition, justice was maintained by informing the participants that the researcher and app developers would have limited access to their dose readings and profiles to ensure those were accurately registered.

### Data Analysis

The verbatim transcripts were analyzed by the researchers using reflective thematic analysis (RTA) described by Braun et al [22]. All the researchers conducting the study hold PhDs with expertise in health science and informatics. The researchers from health science conducted the primary data analysis, as they were experienced in conducting and analyzing qualitative data. Researchers from informatics validated the findings of the data analysis.

This six-phase analytic process includes the following: (1) familiarization with the data, (2) generating initial codes, (3) generating themes, (4) reviewing potential themes, (5) defining and naming the theme, and (6) producing the report. Braun et al [22] explain that RTA concerns the researcher’s reflective and thoughtful engagement with the data and the analytical process [23]. The responses were then labeled as either positive, neutral, or negative [24]. A deductive and inductive approach was used in step 3 of the RTA process. The researchers revisited the data backward and forward. In the deductive approach, hypothesized themes were already in place and based on the researcher’s prior knowledge and existing theory [25]. These include the core functionalities of the mobile app features, namely dose tracking, education, journaling, and mindfulness, upon which the participants needed to reflect. However, in this study, an inductive approach was also necessary to generate new themes from the data. In step 4, the themes were analyzed and dissolved into each other if similarities were found. Data saturation was reached particularly with regard to the user’s perceptions of the usefulness of the app, which were positive. The Results section presents the coding tree used in the data analysis to establish the final themes.

## Results

### Overview

The following coding tree (Table 2) was used to establish the final themes.

**Table 2:** Reflective thematic analysis coding tree used to establish final research themes.

Theme and category		Code
**Theme 1: the usefulness of** PregiDose		
	Dose tracking	Very useful (app) Quite simple (downloading)
	Education	Very nice—education links Informative
	Journaling	Endure the most Express feelings Daily reflection
**Theme 2: the barriers toward adoption and use**		
	Time constraints	Time slot for use High workloads Busy departments
	Physiological conditions of pregnancy	Pregnancy brain Fatigue Exhaustion
**Theme 3: recommendations for the advancement of** PregiDose		
	User support	Weekly information Notifications
	Interest	Milestones
	Automation	Dependence on mobile phones Ease of access Replacement of dosimeter

As depicted in Table 2, three overarching themes were defined as follows: (1) the usefulness of PregiDose, (2) barriers to PregiDose adoption and use, and (3) recommendations for the advancement of PregiDose. The subsequent sections elaborate on the themes with literature control. Individual participant responses were anonymized and demarcated as P1 to P4.

### Usefulness of PregiDose

The Data Analysis section stated that the study initially used a deductive approach to analyze the data. Accordingly, 3 subthemes were hypothesized based on the core functionalities of the mobile app. Hence, the participants were asked how they had experienced these functionalities to assess the usefulness of the app in a real-life setting. The participants’ responses were labeled as positive because they had found the app beneficial, as described in the following narrations:

It was very useful.

P2

**Figure 4:**
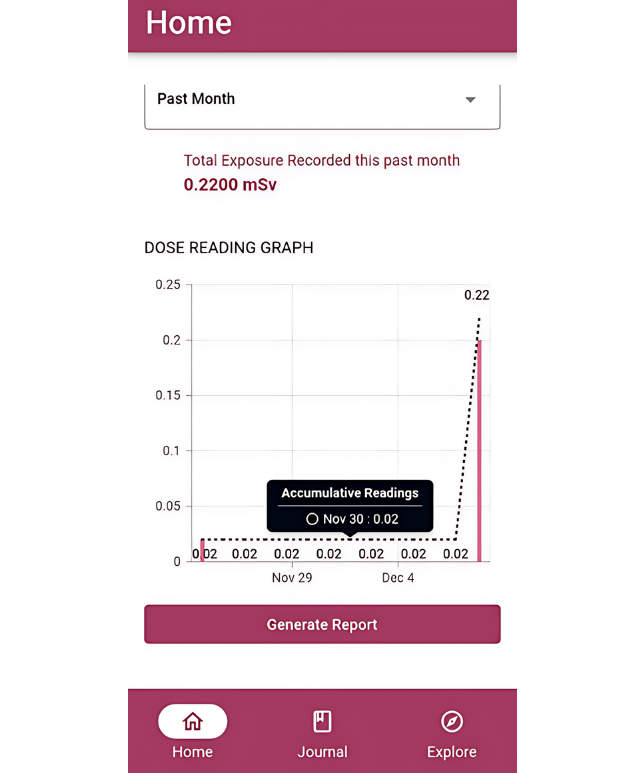
Graph reflecting accumulative fetal radiation doses.

I like the fact that we can see the chart [Figure 4] and the accumulated doses. Usually, in the department we have a paper that we had to write the doses on, it’s a very old school way of doing it.

P4

They further indicated that the educational features were helpful, with statements, such as, “It was very nice” (P1) and *“*It was informative*”* (P2)*.* The participants indicated that although they did not actively engage with the journaling feature, they thought it could potentially be useful, as a participant stated the following:

Radiographers we go through the most, especially with our work. So, we just kept sharing our feelings there [journal], how the day went, and how the work as a whole is going, as a radiographer.

P2

Other participants indicated that they had never practiced manual journaling before and found it difficult to adopt, as seen in this statement:

I haven’t used it, I know some of my colleagues said they wished they had a journaling feature and write down how they were feeling, but I’m not a big journal user, so for me it was not my thing.

P4

Some participants who had indeed engaged with the mindfulness features had labeled it positive, as described in the following narrative:

I think it’s very nice to have some information about other stuff that you can do.

P1

The participants also commented on the technical aspects of the mobile app and viewed downloading and using it positively, as indicated in the following narration:

It was quite simple.

P1

Consequently, the ease of use and simplicity are positive indicators of the usability of the app. However, participants described some technical aspects that hindered the app’s accuracy regarding the accumulative dose and data capturing using the camera. A participant shared real-time screenshots of these challenges to support her contention:

Sometimes it shows as many missing readings, and if I say go to the journal and back it will only be the two or three readings missing. But if I edit the reading, others go missing again.

P1

The participant also reported challenges when trying to capture the data from her dosimeter using the camera function; however, the participant discovered alternate ways to overcome the problem, as reflected in the following narrations:

The bottom icons are in the way and I am unable to take a pic. So have to take it beforehand and then upload it.

P3

These findings suggested that the dose-tracking feature worked effectively, provided the readings were consistently recorded. However, the accuracy of the recorded information was compromised when the participants did not input their doses on a particular day. Therefore, these findings are labeled negative and require intervention from the technical support team.

### Barriers to the Adoption of PregiDose

In this theme, several barriers emerged that apparently influenced the user’s adoption and use of the app negatively, including physiological barriers, motivational barriers, and time constraints. The participants indicated they were tired and forgetful, which resulted in them not engaging with the wellness support features of the app, as described in the following narrative:

We get tired also.

P2

My pregnancy brain, it’s just so difficult to remember.

P1

In keeping with the support features, many participants indicated they “Didn’t see that*”* (mindfulness; P2) and “Didn’t view that” (mindfulness; P3). This oversight might be attributable to a lack of motivation or the persuasive nature of this feature.

Participants indicated that the time constraints due to work responsibilities impacted their ability to engage fully with support features other than the primary dose recording function, described as follows:

The time slot that I use the app is, I put my reminder in for eight o’clock. That’s when I get to work or I’m at work already for a while. So that’s the time that I set out. There’s not really much more time. And when you leave for work, you don’t really think about the app anymore because I see it as a work function.

P3

We can be very busy, so there’s no time.

P2

The participants then provided recommendations for advancing the mobile app based on their experiences using it in a real-life setting.

### Recommendations for the Advancement of PregiDose

In this theme, the participants reflected on strategies that could improve the adoption and use of the app. These strategies included notifications, interest, and automation.

In theme 2, it was evident that the pregnant radiographers’ physiological states can be attributed to radiographers not inputting their daily doses consistently. Consequently, the participants suggested receiving daily reminders from the app, stated as follows:

A notification on the phone where it reminds you to record your dose. It would be very helpful because it will remind us to insert our recording. If you don’t record, it means you’re not doing anything with the app.

P2

Some participants also reported not seeing and engaging with the journaling and mindfulness features. In theme 3, the participants indicated that they perceived the app as a work function and did not feel the need to use it after work hours. Therefore, it is evident that pregnant radiographers require something that would stimulate their interest in using the app beyond its dose-tracking features. The participants suggested the following:

I think something that a lot of people would like to engage with is maybe some weekly information about your baby.

P1

I think maybe some articles, like I am eight weeks, maybe something that tells you that this is week eight, these are the things that will be happening, maybe baby is developing this and that.

P4

Given the barriers described in theme 3, the interviewer probed further on strategies that could be useful for ensuring consistent dose inputting. This included automatized dose recording, whereby the mobile device itself could be used as a dosimeter. The participants responded positively, as evidenced by the following narrations:

I think it would be very great. It would be a very useful feature because now, like our phones, we have our phones everywhere we go, everywhere in the department, wherever we go. So sometimes they say, okay, we are supposed to wear our dosimeters wherever we go and every day, but I feel like a phone is more, compared to a dosimeter, I feel like a phone is much better. You look for your phone, not your dosimeter first.

P2

Yeah. I think so because now you’re walking around with a bag all day with your cell phone, your dosimeter, your other dosimeter, your name tag. So now it would be nice if your cellphone could you replace at least the two dosimeters.

P3

It would be cool if you could connect the dosimeter to the app, like an automated thing.

P4

These views describe the need for automation to advance the app. The Discussion section discusses each theme in relation to the literature.

## Discussion

### Principal Findings

The study adopted a DSR process to inform the development of the app. DSR is inherently pragmatic and thus is concerned with the ability of the developed artifact to solve real-life problems [26]. Jabangwe et al [27] emphasized the importance of adopting an effective development model such that it could serve as a successful high-end mobile app within the context of competitive mobile app development [27]. This aspect is especially significant in the research context, which focuses on creating theories, modeling frameworks, and constructing methods for mobile apps in various settings [28]. Therefore, the themes that emerged in this study reflect the app’s ability to solve real-life problems, on which the upcoming section elaborates.

### Usefulness of PregiDose

The successful adoption of mobile apps depends on the perceived usefulness of the app in the user’s daily life [29]. Vaghefi and Tulu [29] explained that the intervention should align with users’ personal goals to facilitate behavioral change rather than being a goal imposed on them. Traditional methods of fetal dose tracking include writing doses in a logbook or data capture sheet provided by the employer [30]. However, the findings in this theme suggest that pregnant radiographers perceived the app for dose tracking as aligned with the advancements of the technological era. Specifically, the pregnant radiographers in this study found the dose-tracking feature and educational links the most useful. Hughson et al [31] explained that pregnant women prefer using pregnancy apps relevant to their health care context [31]. In this setting, the app was designed to accommodate the needs of a pregnant radiographer working in an ionizing radiation environment. This presents a unique offering in the context of pregnancy apps for pregnant radiographers. Responses to features that support mental well-being, such as journaling, were labeled as neutral and mindfulness as positive. Pregnancy apps are fundamentally based on the idea of self-tracking [32]. However, apps also have the potential to exploit confidential information about the user, such as bodily functions, behaviors, and social relationships [32]. The technical teams overseeing the backend operation of the app have access to these data and might have access to the information entered by the user. Journaling offers the user a platform to share personal thoughts and feelings. Consequently, it is understandable that they might not want to use the app for this function. The findings in this study indicated that the pregnant radiographers welcomed the concept of journaling; however, their disengagement could indicate underlying apprehensions associated with these factors as described by Barassi [32].

### Barriers to the Adoption and Use of PregiDose

A radiography department often encounters high patient volumes and a consequent increased workload for radiographers [33]. Several studies reported that radiographers generally experience physical and emotional burnout because of their highly stressful work environment [34]. Rajan and Dhar [35] further reported that work-related stressors cause fatigue and backache among general radiographers [35]. However, such physical and emotional exhaustion is heightened for pregnant radiographers because of the physiological and somatic changes associated with pregnancy, which manifest in tiredness, slight dyspnea, and backache, among others [36]. Accordingly, the participants’ responses confirm that pregnant radiographers might experience increased fatigue and thus have no energy and desire to interact with a work-related device. In addition to fatigue, the phenomenon of the “pregnancy brain” also emerged. This is a term adopted by the lay public to describe the common occurrence of memory lapses in pregnant women. This might be attributed to the pregnancy hormones remodeling the brain architecture and neural functioning [37]. Thus, the phenomenon aligns with the participants forgetting to engage with the app.

### Recommendations for the Advancement of PregiDose

The study participants recommended strategies, such as notifications, pregnancy information, and automation that could promote behavioral changes regarding the consistency of dose recording and engagement with the well-being features.

Notifications are described as alerts or reminders triggered by the app to remind the user to engage with the app for the intended health goal [29]. Vaghefi and Tulu [29] explained that some users perceive notifications as a distraction, although other users, such as participants in this study, prefer frequent notifications to promote continued use. In addition to notifications, information regarding pregnancy is also deemed beneficial and a promoter of ongoing engagement. A study by Bush et al [38] noted that the most used mobile app features are pregnancy and postpartum health milestone screening. However, a study by Musgrave et al [39] indicated that pregnant women deem fetal movement awareness, pregnancy weight, and breastfeeding features as valuable behavioral change indicators.

Nonetheless, these features are strongly related to pregnant women in general. mHealth apps are developed to meet a targeted behavior change. In the context of this study, the behavior change required is consistent fetal dose recording. Given the availability of a vast array of pregnancy apps and the limited engagement of well-being features of PregiDose, the presumption would be that the PregiDose app can remain within the confines of fetal dose tracking and radiation education.

Finally, the participants recommended automation. Automation is defined as computers or machines operating without the need for human intervention [40]. In the context of mHealth, the concept of Internet of Things (IoT) has grown rapidly in recent years. The IoT constitutes an interconnected network of hardware or software providing automation in collecting health information [41]. Examples of the IoT in health include wireless body area networks and radio-frequency identification [41]. In this study, the participants were still required to input data manually into the mobile app. Hence, the concept of automation could eliminate the prevalence of inconsistent mobile app use, whereby readings are automatically captured without any human action required.

### Future Implementation

As discussed earlier, the end users indicated that although the app was beneficial, it remained a manual process dependent on the user; hence, the participants recommended automation. Furthermore, the idea emerged that the IoT could play a significant role in the future of occupational radiation safety. Rose et al [42] referred to the IoT as scenarios where network connectivity and computing capabilities extend to everyday items not directly associated with computers, allowing these devices to generate, exchange, and consume data with limited human intervention [42]. In the context of this study, objects and items can refer to pocket reading alarm dosimeters. Dosimeters generate radiation dose readings and can also potentially upload data to the mobile app automatically, without intervention from the pregnant radiographers. This IoT was realized by Kim et al [43], who developed a radiation exposure monitoring system for underwater exposure [43]. That device was connected to a mobile app to monitor radiation values generated from the detector [43]. However, Ishigaki et al [44] used an actual mobile device to serve as a radiation detector for non–health workers living in Fukushima, where the Daiichi Nuclear Power Plant accident occurred in 2011 [44]. This application of mobile devices led to several other developments in mobile app technology being used as radiation detectors. Johary et al [45] described how advanced image sensors in smartphone cameras can detect ionizing radiation as well as visible light. Although the measurements are reportedly not as accurate as a dosimeter, they are sufficiently useful to detect radiation levels before they reach threshold limits [45]. The limitations associated with this technology are that smartphones’ heat and battery levels can influence the accuracy of the app. The authors further reported that it is imperative to evaluate the image sensors of commercially available smartphones before they can be used as radiation alarms [45]. Therefore, it is evident that dosimeters and smartphones could serve as IoT devices for radiation dose monitoring.

### Limitations

The radiography workforce comprises a heterogeneous cohort of younger and older male and female health care workers, aged between 21 and 65 years. Hence, the pool of pregnant radiographers is comparatively quite small; therefore, the sample size of this study is limited. However, this qualitative research placed particular importance on rich data, not statistical significance. Although this remains true, the study would have benefited from a larger sample size. Indeed, the researcher attempted to contact the remaining pregnant radiographers using the app; however, they did not respond to the second notification. At this juncture, the researcher reflected on theme 3, namely barriers to adoption, in which pregnant radiographers reported on several factors inhibiting their interaction with the app. Therefore, the study assumed that further engagement through an interview might have been too demanding for the remaining radiographers. These findings align with the limitations described by Karlsen et al [46] and Khakurel et al [47], who also found limitations when evaluating the occupational health and safety apps in a real-life setting. Consequently, the study recommends having a larger cohort of participants, a longer mobile app engagement period, and an alternative time-effective data collection method that would yield more feedback.

### Conclusions

PregiDose is an occupational health and safety mobile app developed for pregnant radiographers through a DSR approach. The study’s findings indicate positive views of the app’s usefulness, whereby the app offers a modern method of dose tracking in keeping with technological advancements in the context of self-tracking. This enables the pregnant radiographer to keep track of her daily doses accurately and thus, ensure the safety of the mother and her unborn child, who are exposed to ionizing radiation environments. Features to promote well-being were also included as supportive add-ons for pregnant radiographers, with the presumption of an all-in-one app. However, given the availability of a vast array of pregnancy apps and the limited engagement of the well-being features in PregiDose, the assumption would be that PregiDose could remain within the confines of fetal dose tracking and radiation education. The study revealed a need for automation, such as using the IoT, within the occupational radiation safety context. This notion introduces several possibilities for future research to explore as far as fetal radiation dose monitoring is concerned.

In summary, PregiDose is a radiation dose-tracking app developed through a DSR strategy. Pregnant radiographers have inconsistent fetal dose-tracking methods and decreased mental and emotional support, identified through the problem awareness step of DSR. The suggestion emanating from the problem awareness was a mobile app to support pregnant radiographers. Three core functional areas were designed using a user-centered approach: dose tracking, education, and wellness. Each feature addressed key challenges experienced by pregnant radiographers working in hazardous ionizing radiation environments. We also concluded that there is a need for automation in fetal dose tracking using the IoT.

## Appendix

Multimedia Appendix 1 PregiDose Promotional video.

## Abbreviations

DSR: design science research
IoT: Internet of Things
mHealth: mobile health
RTA: reflective thematic analysis

## Data Availability

The datasets generated during and analyzed during this study are available from the corresponding author on reasonable request.

## References

[1] Daly LM, Horey D, Middleton PF, Boyle FM, Flenady V (2018). The effect of mobile app interventions on influencing healthy maternal behavior and improving perinatal health outcomes: systematic review. JMIR Mhealth Uhealth.

[2] Etim A, Etim DN, Scott J (2020). Mobile health and telemedicine: awareness, adoption and importance of health study. Int J Healthc Inf Syst Inform.

[3] Shaw R, Stroo M, Fiander C, McMillan K (2020). Selecting mobile health technologies for electronic health record integration: case study. J Med Internet Res.

[4] Lupton D, Pedersen S (2016). An Australian survey of women's use of pregnancy and parenting apps. Women Birth.

[5] Bjelica A, Cetkovic N, Trninic-Pjevic A, Mladenovic-Segedi L (2018). The phenomenon of pregnancy — a psychological view. Ginekol Pol.

[6] Maternal health. World Health Organization.

[7] Sherer MA, Visconti PJ, Ritenour E, Haynes KW (2013). Radiation Protection in Medical Radiography - E-Book.

[8] Fushiki S (2013). Radiation hazards in children - lessons from Chernobyl, Three Mile Island and Fukushima. Brain Dev.

[9] Applegate KE, Findlay Ú, Fraser L, Kinsella Y, Ainsbury L, Bouffler S (2021). Radiation exposures in pregnancy, health effects and risks to the embryo/foetus-information to inform the medical management of the pregnant patient. J Radiol Prot.

[10] FU R, Shen Y, Noguchi H (2024). In utero exposure to radiation fear and birth outcomes: evidence from the Fukushima nuclear power plant accident. SSRN. Preprint posted online on December 1, 2023.

[11] Valentin J (2000). Annals of ICRP: Pregnancy and Medical Radiation.

[12] Essop H, Kekana M, Smuts H, Masenge A (2023). Fetal dosimeter access, usage, and training among pregnant radiographers in South Africa. J Radiol Nurs.

[13] Zhang X, Liu CZ, Choo KK, Alvarado JA (2021). A design science approach to developing an integrated mobile app forensic framework. Comput Secur.

[14] March ST, Smith GF (1995). Design and natural science research on information technology. Decis Support Syst.

[15] Schnall R, Rojas M, Travers J, Brown W III, Bakken S (2014). Use of design science for informing the development of a mobile app for persons living with HIV. AMIA Annu Symp Proc.

[16] Sengupta A, Subramanian H (2022). User control of personal mHealth data using a mobile blockchain app: design science perspective. JMIR Mhealth Uhealth.

[17] Alharbey R, Chatterjee S (2019). An mHealth assistive system "MyLung" to empower patients with chronic obstructive pulmonary disease: design science research. JMIR Form Res.

[18] Mdletshe S, Oliveira M, Twala B (2021). Enhancing medical radiation science education through a design science research methodology. J Med Imaging Radiat Sci.

[19] Vaishnavi V, Kuechler B, Duraisamy s Design science research in information systems. Association for Information Systems.

[20] Essop H, Kekana R, Smuts H (2024). Co-designing of a prototype mobile application for fetal radiation dose monitoring among pregnant radiographers using a design thinking approach. Health Informatics J.

[21] Sommerville I (2011). Software Engineering Ninth Edition.

[22] Braun V, Clarke V, Hayfield N (2019). ‘A starting point for your journey, not a map’: Nikki Hayfield in conversation with Virginia Braun and Victoria Clarke about thematic analysis. Qual Res Psychol.

[23] Byrne D (2021). A worked example of Braun and Clarke’s approach to reflexive thematic analysis. Qual Quant.

[24] Wang CJ, Chaovalit P, Pongnumkul S (2018). A breastfeed-promoting mobile app intervention: usability and usefulness study. JMIR Mhealth Uhealth.

[25] Proudfoot K (2022). Inductive/deductive hybrid thematic analysis in mixed methods research. J Mix Methods Res.

[26] Mdletshe S, Motshweneng OS, Oliveira M, Twala B (2023). Design science research application in medical radiation science education: a case study on the evaluation of a developed artifact. J Med Imaging Radiat Sci.

[27] Jabangwe R, Edison H, Duc AN (2018). Software engineering process models for mobile app development: a systematic literature review. J Syst Softw.

[28] Weichbroth P (2020). Usability of mobile applications: a systematic literature study. IEEE Access.

[29] Vaghefi I, Tulu B (2019). The continued use of mobile health apps: insights from a longitudinal study. JMIR Mhealth Uhealth.

[30] Singh T (2003). Pregnancy in the workplace: an overview of legislation and current debates. S Afr Radiogr.

[31] Hughson JA, Daly JO, Woodward-Kron R, Hajek J, Story D (2018). The rise of pregnancy apps and the implications for culturally and linguistically diverse women: narrative review. JMIR Mhealth Uhealth.

[32] Barassi V (2017). BabyVeillance? Expecting parents, online surveillance and the cultural specificity of pregnancy apps. Soc Media Society.

[33] Robertson S, Olanloye EE, Hon Y, England A, McNair H, Cruickshank S (2022). Are radiographers suffering from symptoms of compassion fatigue due to occupational stress: a systematic review. Radiography (Lond).

[34] Alakhras M, Al-Mousa DS, Lewis S (2022). Assessment and correlation between job satisfaction and burnout among radiographers. Radiography (Lond).

[35] Rajan D, Dhar P (2023). Health problems among radiographers: an empirical study in private hospitals. Health Econ Manag Rev.

[36] Malik A, Suresh S, Sharma S (2017). Factors influencing consumers’ attitude towards adoption and continuous use of mobile applications: a conceptual model. Procedia Comput Sci.

[37] Brown E, Schaffir J (2019). "Pregnancy brain": a review of cognitive changes in pregnancy and postpartum. Obstet Gynecol Surv.

[38] Bush NE, Skopp N, Smolenski D, Crumpton R, Fairall J (2013). Behavioral screening measures delivered with a smartphone app: psychometric properties and user preference. J Nerv Ment Dis.

[39] Musgrave LM, Kizirian NV, Homer CS, Gordon A (2020). Mobile phone apps in Australia for improving pregnancy outcomes: systematic search on app stores. JMIR Mhealth Uhealth.

[40] Parasuraman R, Mouloua M, Molloy R, Hilburn B, Parasuraman R, Mouloua M (1996). Monitoring of automated systems. Automation and Human Performance.

[41] Haghi Kashani M, Madanipour M, Nikravan M, Asghari P, Mahdipour E (2021). A systematic review of IoT in healthcare: applications, techniques, and trends. J Netw Comput Appl.

[42] Rose K, Eldridge S, Chapin L (2015). The internet of things: an overview. Internet Soc (ISOC).

[43] Kim JH, Park KH, Joo KS (2018). Development of low-cost, compact, real-time, and wireless radiation monitoring system in underwater environment. Nucl Eng Technol.

[44] Ishigaki Y, Matsumoto Y, Ichimiya R, Tanaka K (2013). Development of mobile radiation monitoring system utilizing smartphone and its field tests in Fukushima. IEEE Sensors J.

[45] Johary YH, Trapp J, Aamry A, Aamri H, Tamam N, Sulieman A (2021). The suitability of smartphone camera sensors for detecting radiation. Sci Rep.

[46] Karlsen IL, Svendsen PA, Abildgaard JS (2022). A review of smartphone applications designed to improve occupational health, safety, and well-being at workplaces. BMC Public Health.

[47] Khakurel J, Melkas H, Porras J (2018). Tapping into the wearable device revolution in the work environment: a systematic review. Inf Technol People.

